# Therapeutic modulation of gene expression in the disease state: Treatment strategies and approaches for the development of next-generation of the epigenetic drugs

**DOI:** 10.3389/fbioe.2022.1035543

**Published:** 2022-10-17

**Authors:** Joseph Rittiner, Mohanapriya Cumaran, Sahil Malhotra, Boris Kantor

**Affiliations:** ^1^ Department of Neurobiology, Duke University Medical Center, Durham, NC, United States; ^2^ Viral Vector Core, Duke University Medical Center, Durham, NC, United States; ^3^ Duke Center for Advanced Genomic Technologies, Durham, NC, United States

**Keywords:** adeno-associated vector (AAV), lentiviral (LV) vector, epigenome-editing technology, CRISPR (clustered regularly interspaced short palindromic repeat)/Cas9 (CRISPR associated protein 9)-mediated genome editing, transcriptional repressor

## Abstract

Epigenetic dysregulation is an important determinant of many pathological conditions and diseases. Designer molecules that can specifically target endogenous DNA sequences provide a means to therapeutically modulate gene function. The prokaryote-derived CRISPR/Cas editing systems have transformed our ability to manipulate the expression program of genes through specific DNA and RNA targeting in living cells and tissues. The simplicity, utility, and robustness of this technology have revolutionized epigenome editing for research and translational medicine. Initial success has inspired efforts to discover new systems for targeting and manipulating nucleic acids on the epigenetic level. The evolution of nuclease-inactive and RNA-targeting Cas proteins fused to a plethora of effector proteins to regulate gene expression, epigenetic modifications and chromatin interactions opened up an unprecedented level of possibilities for the development of “next-generation” gene therapy therapeutics. The rational design and construction of different types of designer molecules paired with viral-mediated gene-to-cell transfers, specifically using lentiviral vectors (LVs) and adeno-associated vectors (AAVs) are reviewed in this paper. Furthermore, we explore and discuss the potential of these molecules as therapeutic modulators of endogenous gene function, focusing on modulation by stable gene modification and by regulation of gene transcription. Notwithstanding the speedy progress of CRISPR/Cas-based gene therapy products, multiple challenges outlined by undesirable off-target effects, oncogenicity and other virus-induced toxicities could derail the successful translation of these new modalities. Here, we review how CRISPR/Cas—based gene therapy is translated from research-grade technological system to therapeutic modality, paying particular attention to the therapeutic flow from engineering sophisticated genome and epigenome-editing transgenes to delivery vehicles throughout efficient and safe manufacturing and administration of the gene therapy regimens. In addition, the potential solutions to some of the obstacles facing successful CRISPR/Cas utility in the clinical research are discussed in this review. We believe, that circumventing these challenges will be essential for advancing CRISPR/Cas-based tools towards clinical use in gene and cell therapies.

## Introduction

### Epigenetics overview of the components, and its involvement in the regulation of gene expression

**FIGURE 1:**
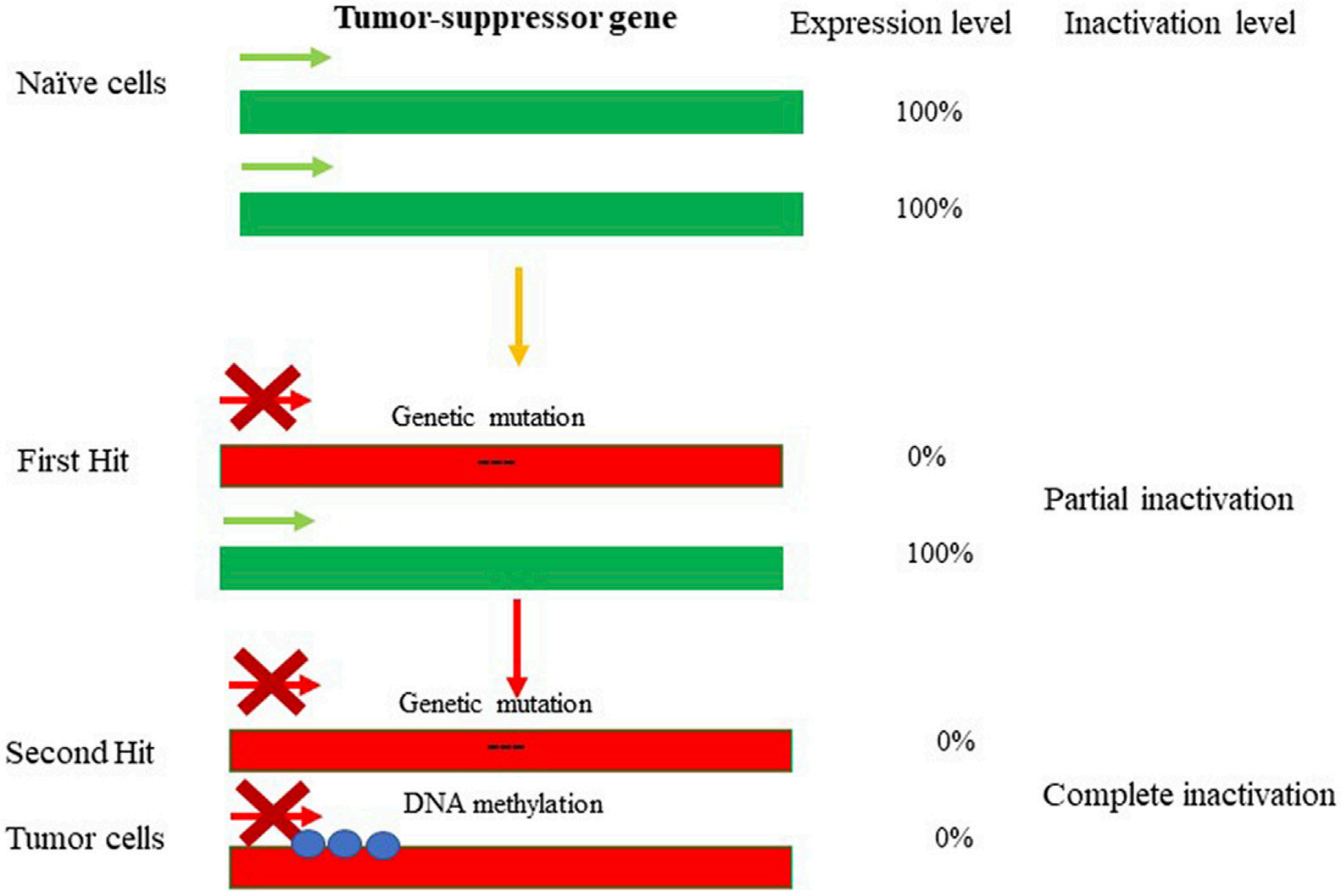
Genetic and epigenetic inactivation of tumor-suppressor genes (TSGs). Mutations lead to inactivation of one allele of tumor-suppressor gene resulting in partial loss-of-function. The first hit highlights the event leading to partial loss of gene expression of the TSG. The selection pressure then could promote the second hit resulting in epugenetically-driven complete loss-of-function. The inactivity of TSG leads to tumorigenesis.

The term “epigenetics” was introduced by Conrad Waddington in the early 1940s to define “the branch of biology which studies the causal interactions between genes and their products which bring the phenotype into being” (Waddington, 2012). The field of epigenetics in its common view includes a wide range of heritable and reversible changes in gene expression and regulation that occur in response to external (epi) environmental inputs and that do not result from alterations in nucleotide sequence of the DNA. The epigenetic marks typically arise *via* DNA methylation, DNA-binding repressor machineries, chromatin remodeling and post-translational histone modifications (PTHMs) (Razin and Riggs, 1980), (Razin, 1998), (Razin and Kantor, 2005). Epigenetic alterations contrast directly with genetic alterations, including nucleotide substitutions, mutations, insertions/deletions (InDels), site-directed recombination, rearrangements, and virus- or retrotransposon-mediated integration, which permanently and irretrievably change gene structure (reviewed in (Burns, 2020), (Weiss, 2016), (Koboldt et al., 2013)). Nevertheless, genetics and epigenetics are inextricably linked in many ways: epigenetic changes can cause mutations in genes, and conversely, mutations are frequently observed in genes that modify the epigenome. The interplay between genetics and epigenetics in the disease state has been shown for many pathological conditions, including cancer (Figure 1). For example, during tumorigenesis, tumor-suppressor genes (TSGs) can be inactivated through mutations, after which their activity becomes disabled. If the second allele is switched off in a somatically heritable fashion by epigenetic changes, that would lead to complete inactivation of the gene function (a condition known as loss-of-heterozygosity (Baylin and Jones, 2016)). On the other hand, during carcinogenesis, protooncogenes can become over-activated in such a fashion that enhances the pace of cell division or prevents cell death: the process known as oncogenicity. In this case as well, loss-of-heterozygosity can be caused by imprinting, with the second allele being switched on or off by epigenetic alterations. It has to be noted that aberrant expression of oncogenes caused by genetic mutations or epigenetic alterations are often dominant and drive the formation of cancers. On the other hand, genetic mutations or epigenetic repression of TSGs are often recessive, needing disruptive events in both alleles of a gene for the full manifestation of the transformed phenotype (Baylin and Jones, 2016) and Figure 1. As such, the latter scenario is usually associated with a slower rate of cancer development and progression and generally is less common (Zhang et al., 2020). More directly, somatic duplications and genomic insertions can affect the epigenetic landscape and expression of the affected loci. For instance, transposable elements (TE) or viral genomic insertions could lead to large epigenetic changes resulting in dysregulation of gene expression and function (reviewed in (Misiak et al., 2019)). Here the interplay between genetics and epigenetics is usually bidirectional, exemplified by mutations in the DNMT3B gene, responsible for methylating highly repetitive minor satellite repeats; DNMT3B is considered the major *de novo* DNA methyltransferase (DNMT) in early development owing to its high expression levels (Moarefi and Chedin, 2011). Mutations in DNMT3B cause immunodeficiency, centromeric region instability, and facial anomalies syndrome (ICF; OMIM 242860), a rare autosomal recessive disorder (Okano et al., 1999; Hansen et al., 1999). Lymphocytes from ICF patients show centromeric instability due to hypomethylation at classical satellites 2 and 3 and at the pericentromeric regions of chromosomes, and regional destabilization in gene expression (Xu et al., 1999). Furthermore, the DNA methylation deficiency characteristic to ICF syndrome is associated with an extraordinary collection of chromosomal anomalies, specifically in the vicinity of the centromeres of chromosomes 1 and 16 (Chr1 and Chr16). These aberrations include decondensation of centromere-adjacent (qh) heterochromatin, multiradial chromosomes with up to 12 arms, and whole-arm deletions and translocations. The latter could lead to pathological rearrangement of the chromatin landscape of the affected genomic region associated with global dysregulation of gene expression (Tuck-Muller et al., 2000; Sawyer et al., 2019). Furthermore, pathological overexpression of TEs has been found to cause malfunctioning of immune-inflammatory responses in the brain, and compromising immunity against exogenous infections (reviewed in (Misiak et al., 2019)). The aberrant epigenetic regulation and expression of TEs emerged as a potential mechanism underlying the development of various mental disorders, including autism spectrum disorders (ASD), schizophrenia, bipolar disorder, major depression, and Alzheimer’s disease (AD). As with endogenous retrotransposons and other repetitive elements, exogenous agents (e.g., viruses) have the capacity to alter the genetics and epigenetics of the region-of-integration. In fact, the high capability of retroviruses to integrate into host cell chromosomes raises the possibility of insertional mutagenesis and oncogene activation. Both these phenomena are well known in the interactions of certain types of wild-type (wt) retroviruses with their hosts (Kantor et al., 2014a). More recently, the same phenomenon has been demonstrated with recombinant retroviral vectors (rRVs) used for gene-to-cell transfer, in both animal models and human clinical trials. The oncogenic potential of retroviral vectors materialized in the clinical trial for X-linked severe combined immunodeficiency (X-SCID), as in this trial 2 out of 10 patients developed T cell leukemia as a consequence of the treatment. It has been demonstrated that the integration of retroviral vector genome in the vicinity of proto-oncogene LMO2 in the leukemia patients is what caused the abberant gene expression and subsequent tumorigenicity (Cavazzana-Calvo et al., 2000; Hacein-Bey-Abina et al., 2003a). Similar to gamma-retroviruses, lentiviral vectors are capable of integrating into the host genome, thus potentially retaining the ability to induce onco- and tumorigenicity (Kantor et al., 2014b). Nevertheless, there is no evidence in support of lentiviral vector-mediated oncogenicity; this is possibly related to the presumption that the risk of insertional mutagenesis in nondividing cells is not as large as in dividing cells. As in above examples, disease-causing genetic mutations can affect chromatin in *trans* or have a *cis* effect in altering chromatin configuration. Alternatively, disease may be caused by direct changes in epigenetic marks, such as DNA methylation, commonly found to affect gene regulation. Here is a comprehensive overview of chromatin organization of the genome in health and disease states. Epigenetic modifications are responsible for regulation of gene expression programs in a cell. These modifications can be reversible to some degree, but overall are somatically stable and heritable, such that a parental cell gives rise to daughter cell programmed to have the same epigenetic markers of gene expression after it divides. In the case of nondividing cells, e.g., neurons of the central nervous system (CNS), the epigenetic patterns controlling gene expression are less stable than those of dividing cells and engage in variety of reproducible responses within individual neurons to defined stimuli (reviewed in (Lomvardas and Maniatis, 2016)). The epigenetic profile is defined and as the ensemble of DNA methylation, post-translation histone modifications, histone variants, and chromatin remodelers.

**FIGURE 2:**
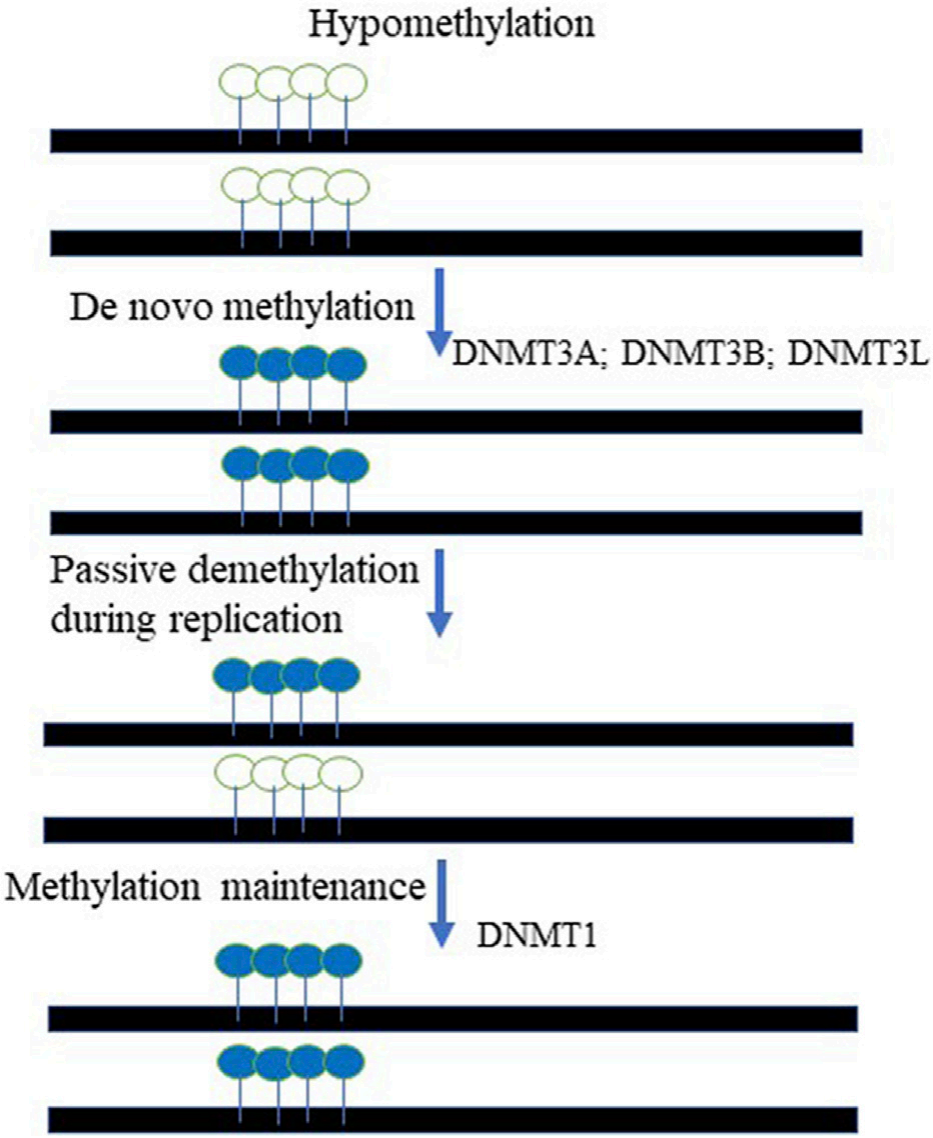
The cycle of DNA methylation. The *De novo* methyltransferases, DNMT3A and DNMT3B paired with DNMT3L chaperon activity methylate unmethylated DNA templates at the CpG context. The maintenance mechanism ensures that hemi-methylated templated created during DNA replication are to be fully methylated on both DNA strands. DNMT1- DNA maintenance methyltransferase is responsible for that activity. Open lollipops represent unmethylated DNA, while painted lollipops represent the DNA that methylated.

In mammals, DNA methylation, occurs predominantly at the carbon-5 position of the symmetrical CpG sequences (5 mC). Due to this, it was proposed decades ago that a mechanism was in place to recognize the hemimethylated CpG site following DNA replication and accurately methylate the unmethylated daughter strand; elegant experiments have subsequently validated this hypothesis (Razin and Riggs, 1980; Razin and Kantor, 2005). In fact, it was demonstrated that DNA methylation is maintained through cell division *via* the activity of DNA methyltransferase 1 (DNMT1), which methylates hemimethylated CpG dinucleotides in daughter cells (Razin and Kantor, 2005; Li et al., 1992). DNA methylation is of paramount importance for mammalian embryonic development. In support of this statement, it has been demonstrated that DNMT1-deficient mice exhibit severe developmental abnormalities, climaxing in early embryonic lethality (Okano et al., 1999; Razin and Kantor, 2005; Li et al., 1992). DNA methylation carries out numerous essential functions within the cell: 5 mC has been implicated in the repression of transposons, repetitive elements and other retroviral-derived sequences, exogenous agents such as viruses and bacteria, and genes. Significantly, DNA methylation has been implicated in the classical epigenetic phenomena of genomic imprinting (Kantor et al., 2006) and X-chromosome inactivation (XCI) (Razin and Riggs, 1980; Mohandas et al., 1981; Lock et al., 1986; Lock et al., 1987; Park and Chapman, 1994). It is important to note that despite its ancient origin, 5 mC methylation has been lost in several eukaryotic lineages, including *Drosophila melanogaster* (Urieli-Shoval et al., 1982), *Caenorhabditis elegans* (Simpson et al., 1986), and others (Zemach and Zilberman, 2010). The biological rationale behind this phenomenon is that DNA methylation at the C residue comes at a cost: 5 mCs are at high risk of undergoing deamination, leading to C → T transitions (Holliday and Grigg, 1993). Thus, organisms with CpG methylation also have reduced CpG content (Razin and Riggs, 1980), (Razin and Kantor, 2005). In addition, many viruses and endogenous retroviral sequences demonstrate relatively low CpG content, as a mechanism to escape repressive chromatin formation. In fact, lentiviral and retroviral vectors generally have low CpG content in the promoter region due to evolutionally-based cytosine deamination (Kantor et al., 2009). The remaining CpGs are largely hypomethylated, thus supporting transcriptional activity of the viruses (Kantor et al., 2009). As mentioned above, DNA methylation serves as the key mechanism regulating expression of the imprinted genes. In fact, aberrant DNA methylation may cause imprinting deficits, resulting in neurological diseases such as Prader-Willi and Angelman syndromes (Kantor et al., 2006). In addition to genomic imprinting and XCI, DNA methylation has a key role in silencing transposons (Arand et al., 2012), (Walsh et al., 1998) and germline-specific genes (Borgel et al., 2010). DNA methylation is also highly enriched in satellite repeats located in the pericentromeric regions (Lewis et al., 1992) and in the bodies of actively transcribed genes (Lister et al., 2009), though the precise function of DNA methylation in both of these contexts is largely unknown. There are two steps in the process of methylating DNA substrates: establishment—a process catalyzed by *de novo* methyltransferases 3A and 3B (DNMT3A and DNMT3B), and maintenance, mentioned above, which is performed by the maintenance methyltransferase I (DNMT1) (Razin and Riggs, 1980), (Razin, 1998), (Razin and Kantor, 2005). There is also a catalytically-inactive form of DNA methyltransferase, DNMT3L, which stably interacts with *de novo* methyltransferases and stimulates their activity **(** Figure 2). The protein readers involved in the methylation process are the methyl-CpG-binding domain (MBD) proteins (Ooi et al., 2007). (Meehan et al., 1989). Up to date, five MBD proteins have been discovered and characterized. Those include MBD1, MBD2, MBD3, MBD4, and methyl-CpG-binding protein 2 (MeCP2). It has been demonstrated that all MBDs interact with nucleosome remodeling, histone-modifying, and histone deacetylase complexes, which leads to gene silencing (Nan et al., 1998; Ng et al., 1999; Kantor et al., 2003) and reviewed in (Razin and Kantor, 2005).

As highlighted above, DNA methylation contributes to heterochromatin formation throughout the recruitment of chromatin remodelers and modifiers, and *via* interaction with the enzymes catalyzing post-translational histone changes (Razin, 1998; Razin and Kantor, 2005). Chromatin modifications involve covalent posttranslational modifications of mostly the protruding N-terminal histone tails with a variety of chemical groups, including methyl, acetyl, phosphate, ubiquitin, and SUMO groups bearing SUMO-interaction motifs (SIMs). The posttranscription histone modifications are off the scope of the current review; we would like to refer the reader to the review by Maze and colleagues for the comprehensive discussion on the topic (Maze et al., 2014).

Interestingly, the same rules governing epigenetic organization of DNA are commonly applied towards human viruses and vectors (reviewed in (Kantor et al., 2014a)). Even more striking is that several studies have shown that the genomes of RNA and DNA viruses, (integrating and non-integrating) including HIV-1, SV40, EBV, HSV, AAV, and HBV, are organized into chromatin structures, which have major effects on viral gene expression and life cycle (Ambrose et al., 1990; Bock et al., 2001; Day et al., 2007; Monahan et al., 2010; Johnson et al., 2011; Kantor et al., 2009). On the same note, a number of research groups focusing on the early stages of the herpes viral life cycle have described a competition between cellular epigenetic silencing of viral genes and viral inhibition of HDAC activity mediated by ICP0 and Us3, as well as IE1 and IE2 of HSV1 and CMV, respectively, to enable the expression of viral genes (Nevels et al., 2004; Poon et al., 2006). In analogy to this phenomenon, we showed that histone deacetylase inhibitors (HDAC*i*) may significantly upregulate gene expression from integrase-deficient lentiviral vector (IDLV) following transduction of various cell lines (Kantor et al., 2009). Consistent with these observations, the increase in viral expression has resulted in a significant decrease in trimethylated H3-K9, typical of silent chromatin (Kantor et al., 2009). This is consistent with the concept that H3-K9 methylation serves as a binding site for HP1; in turn HP1 recruitment leads to gene silencing by recruiting DNA methyltransferases to the targeted genes (Machida et al., 2018). Significantly, four H3-K9 methyltransferase enzymes, G9a and GLP (responsible for mono- and di-methylation), and SUV39h1 and SUV39h2 (responsible for tri-methylation) have also been linked to the silencing of specific genes located in euchromatin (Tachibana et al., 2005), (Peters et al., 2003), (Rice et al., 2003). Conversely, methylation of histone H3-K27 has been linked to several silencing phenomena including homeotic-gene silencing, X inactivation, and genomic imprinting (Cao and Zhang, 2004). As such, it appears that similar or even identical modifications may play different roles in the different spatial contexts. Importantly, some recent studies have challenged the generality of the association between methylated H3-K9 and gene silencing, as they report that in several cell lines the coding regions of a number of active genes are enriched in H3-K9-trimethyl groups and the HP1γ isoform (Vakoc et al., 2005; Lomberk et al., 2006). As highlighted above, DNA methylation and post-translational histone modifications play a key role in providing the foundation by which localized transcription factors support the binding of basic transcriptional machinery. The discovery of DNA methylation and histone modifications in the 1960s, specifically H3/H4-K-acetylation and H3/H4-K methylation, provide a mechanistic insight and validation on the hypothesis on the role of epigenetic modification and the chromatin organization in gene activation and silencing (reviewed in (Razin and Riggs, 1980; Razin, 1998)). However, it was not until the early-2000s that the first direct evidence linking the epigenome and gene regulation by histone modification was reported (Goldberg et al., 2007). More specifically, it has been demonstrated that the yeast SAGA complex contains the Gcn5 histone acetyltransferase (HAT), which serves as well-defined coactivator of gene expression (Huisinga and Pugh, 2004). H3/H4-K acetylation has been shown to be linked with decompressing chromatin structure, which is a feature typical of high-level regulation of gene expression. Discovery of HDAC proteins further solidified the idea that dynamic histone acetylation/deacetylation cycles may regulate gene expression by reversibly decompacting or compacting nucleosomes to allow RNA polymerases to pass through or be blocked off, respectively (Henikoff and Shilatifard, 2011). The network of acetyltransferases and deacetylases is very extensive; there are a number of well-defined and characterized acetyltransferases responsible for histone acetylation including p300 and others (Nakatani, 2001). Similarly, there are at least 18 well-characterized histone deacetylases, divided into four classes, Class I (HDAC1, 2, 3, and 8), Class II (HDAC4, 5, 6, 7, 9, and 10), Class III (the Sirtuins), and Class IV (HDAC 11) (Li and Seto, 2016). More recently, additional reports linked methylation of H3K4, H3K27, H3K36, and H3K79 and others, and the addition of the histone variants H2A.Z and H3.3 and others, as to be defined marks of active or silenced transcription (Razin and Riggs, 1980; Razin, 1998; Henikoff and Shilatifard, 2011; Turner, 2002). As mentioned above, other components of the chromatin that are directly or indirectly recruited by transcription factors are chromatin modifiers and remodelers, which are capable of repositioning and mobilizing nucleosomes to arrange nucleosome-depleted or nucleosome-dense regions where general transcription machinery could bind and initiate or block RNA PolII recruitment, respectively. Many functional properties of histone modifications are inferred from associating their patterns with transcriptional states of loci nearby. For example, as mentioned above, the random inactivation of the X chromosome by the long non-coding RNA Xist involves targeted recruitment of a PRC2 complex which trimethylates H3K27 on one of the two X chromosomes resulting in its inactivation—which is the central event in developmental silencing (Steffen and Ringrose, 2014). This example illustrates how a single histone modification (H3K27me3) is involved in repressing gene expression.

### Epigenetic treatments and drugs

Drugs that interfere with pathological changes in the epigenome have the potential, in principle, of reversing the defined pathophysiological phenotypes in cancer and other diseases stemming from gene dysfunctions—a prospect that has driven development of small molecule drugs targeting DNA methylation- and PTHM-enzymes and other chromatin proteins. For example, deoxycytidine analogs display distinct toxicity in malignant cells by incorporating into DNA and preventing release of DNMT1 after it forms a covalent intermediate with DNA, thus depleting the enzyme (Issa and Kantarjian, 2009). In fact, relatively low doses of the drugs have been demonstrated to be effective in the treatment of myelodysplastic syndrome (Christman, 2002; Li and Seto, 2016; Licht, 2015). As mentioned above, HATs and HDACs play central roles in the pathologies of cancer and other diseases. As such, it is not surprising that a plethora of broadly acting HAT and HDAC inhibitors have been used in the clinic to treat many diseases including cancer (reviewed in (Goldberg et al., 2007)). Notwithstanding some successes achieved using HAT/HDAC inhibition to treat cancer and other diseases, the effect of these therapies is limited and compromised by substantial side effects driven by off-target actions of the drugs. In fact, it is well known that nearly all of the HAT and HDAC inhibitors target non-histone substrates as well as the histones. For example, p300/CBP HAT-acetylases catalyze acetylation of the p53 transcription factor, which could cause up-regulation of the p53-positive feedback loop (Gu and Roeder, 1997), which is a concerning outcome complicating the anti-cancer activity of HAT and HDAC inhibitors. On the same note, about 2000 genes and proteins are regulated by HDACs, which is further complicates the interpretation of the therapeutic effect of HDAC inhibitors. In addition, many small molecule drugs in clinical trials targeting specific histone methyltransferases and demethylases seem likely to inhibit their activities non-specifically, by damaging chromatin in rapidly dividing cells. In fact, there may be no clear cut distinction between “epigenetic drugs” and traditional anti-cancer compounds; an example is doxorubicin and other anthracycline molecules, which have been commonly used in the clinic for decades and are known to cause sizable nucleosome eviction (Pang et al., 2013). All this evidence significantly limits the use of epigenetic drugs, as they greatly compromise cellular memory, gene regulation and genomic stability of normal cells.

### CRISPR/Cas systems: Introduction and overview

A more specific and precise strategy aimed to modulate a pathological state of epigenomics could be achieved *via* gene therapy approach. The current development of this clinical field holds a significant promise of treating epigenetic, and transcriptomic aberrations, as well as disease-causing genetics (Kantor et al., 2014b). Indeed, gene therapy has been proven to be promising treatment option for a number of hereditary diseases and conditions, including but not limited to neurological disorders. It has to be pointed out that the potential benefits of using gene therapy approach paired with viral delivery platforms for correcting these diseases are enormous, and as a consequence extensive effort have been made to evolve and optimize viral systems for gene-mediated transfer targeting the central nervous system (CNS). Fortuitously, the arsenal of gene therapy therapeutics has been tremendously expended and shaped with the evolution of highly innovative genome and transcriptome engineering tools based on the CRISPR/Cas RNA-guided nuclease systems, which have transformed our ability to precisely manipulate nucleic acids (Rittiner et al., 2020). Importantly, CRISPR/Cas-based gene therapy products have enabled precision and fine-tuned alteration of gene transcriptomics in sustained, reliable and long-term modes (Dong and Kantor, 2021). The goal of this review is to comprehensively outline the CRISPR/Cas toolkit, paired with viral vectors for gene therapy application in the CNS and as a platform-of-choice for the development of next generation of therapeutics to combat NDDs. As such, we aim in the below session to provide a general introduction and overview of the CRISPR/Cas systems. The CRISPR/Cas, clustered regularly interspaced short palindromic repeats/CRISPR-associated proteins in nature acts as a prokaryotic acquired-immunity mechanism that evolved to target and destroy foreign DNAs and RNAs of phages, archaea and viruses (Barrangou et al., 2007; Horvath et al., 2008; Sorek et al., 2008). The system could combine a variety of components, which may widely differ structurally and functionally (reviewed in (Koonin et al., 2017; Agarwal and Gupta, 2021)). Despite the diversity, all CRISPR/Cas systems share a CRISPR RNA component [guide RNA (gRNA) and trans-activating RNA (tracrRNA)] which defines targeting specificity (Jinek et al., 2012). The simplicity of class II CRISPR/Cas system which fit the viral-based delivery platforms is extremely appealing for gene-editing applications in humans (reviewed in (Rittiner et al., 2020)). Critcally, CRISPR/Cas9 acts as a single effector protein; in contrast, the class I CRISPR/Cas enzymes are only active in the context of multi-subunit protein complexes (reviewed in (Rittiner et al., 2020)). To create a more compact system the two RNA components have been combined into a single guide RNA (sgRNA), expressed from general Pol III promoters such as U6, 7SK, or H1 (Cong et al., 2013; Ran et al., 2013). The protein component of the class II CRISPR/Cas system—Cas9—paired with gRNA recognizes and binds to a specific sequence within the targeted DNA, known as its protospacer-adjacent motif (PAM). The binding triggers DNA unwinding, followed by hybridization of the gRNA and the exposed DNA strand (the protospacer) (Cong et al., 2013; Ran et al., 2013). After full Watson-Crick base pairing between gRNA and protospacer, the two nuclease domains of catalytically active Cas9 will cut each strand of the targeted dsDNA, causing a double-strand DNA break (DSB) (Cong et al., 2013; Ran et al., 2013). The DSB could activate a non-homologous end joining, NHEJ-mediated DNA repair machinery responsible for trimming and re-ligating the damaged ends. This repair process is prone to forming small deletions or insertions (InDels) that can be harnessed to create gene knockouts (Jinek et al., 2012; Mali et al., 2013; Cong et al., 2013). For example, targeting a coding sequence located within exon, InDel-mediated frameshifts may result in the creation of premature stop codons downstream of the targeted loci, which would disrupt protein translation. On the other hand, dual-gRNA targeting of two separated genomic loci may be useful in creating large deletions in the desired DNA loci (Cong et al., 2013; Ran et al., 2013; Zhu et al., 2016), including megabase-size deletions (Essletzbichler et al., 2014). The ability of CRISPR/Cas to introduce insertions in the DNA sequences has been widely explored as well. For example, Cas9-mediated and NHEJ-mediated gene tagging strategies have been developed based on the integration of linear DNA oligonucleotides at nuclease-cleavage sites. This approach has been further developed for homology-independent targeted integration (HITI), utilizing a tag flanked with gRNA target sites, so that Cas9 can simultaneously release it from a plasmid and cleave a recipient genomic target adjacent to a gene-of-interest (Suzuki et al., 2016). It has to be noted that the error-prone NHEJ-mediated repair is the predominant mechanism to address DSBs in eukaryotes; alternatively, a repair template with homology to the target site can be delivered with Cas9 to stimulate the error-free homology-directed repair (HDR) process, but typically at a far lower efficiency than the former (reviewed in (Wyman and Kanaar, 2006; Rittiner et al., 2020)). HDR mechanism can be utilized to create a specific alteration in the genomic site, such as a point mutation or insertion of a longer fragment of DNA (Wyman and Kanaar, 2006; Rittiner et al., 2020)). Increasing the efficiency of HDR following nuclease-mediated DNA breakage is widely pursued to fully harness the power of genome editing to introduce precise genomic alterations.

As mentioned above, the Cas9 protein is capable of binding to the target DNA provided it recognizes a PAM motif (reviewed in (Rittiner et al., 2020)). However, while the PAM requirement is necessary for specific interaction between CRISPR/Cas components and target DNA, it is also a constraint. For instance, the canonical PAM associated with the Cas9 nuclease of *Streptococcus* pyogenes (SpCas9) is the sequence 5′-NGG-3′ (Anders et al., 2014). A tremendous amount of effort has been put into the discovery of novel variants of Cas, particularly Cas9, to diversify available PAM compositions. In fact, to increase coverage of potential target sites, various rational engineering approaches have been applied, resulting in the creation of novel Cas9 isoforms with altered PAM specificities (we refer the reader for the detailed discussion on the topic to our review by Rittiner and colleagues ((Rittiner et al., 2020)). Similarly, other classes of Cas endonucleases are out-of-scope of this review and are comprehensively covered in the above review article. Briefly, Kleinstiver and colleagues used enhanced selection screening in bacteria to identify new mutants of SpCas9. The effort resulted in discovery of three variants (VQR, EQR, and VRER) that recognize the novel PAM sequences NGAN/NGNG, NGAG, and NGCG, respectively (Kleinstiver et al., 2015a; Kleinstiver et al., 2015b). Furthermore, Hirano and colleagues evolved Cas9 from Francisella novicida, which has been engineered to recognize a non-canonical 5′-YG-3′ PAM (Hirano et al., 2016a; Hirano et al., 2016b). However, this Cas9 is one of the largest members of Cas9 family (Chylinski et al., 2013). In fact, FnCas9 consists of 1,629 amino acids and is significantly larger than other Cas9 orthologs such as SpCas9 (1,368 amino acids) and SaCas9 (1,053 amino acids) (reviewed (Rittiner et al., 2020)). Most recently, two significant SpCas variants were engineered: SpG, which is capable of targeting an expanded set of NGN PAMs, and a near-PAMless variant called SpRY (Walton et al., 2020). Collectively, SpG and SpRY enable unconstrained targeting using CRISPR-Cas9 nucleases across nearly the entire genome, with single base-pair precision (Walton et al., 2020). Using SpRY, the authors were able to correct mutations associated with human diseases located in previously “un-editable” regions of the genome (Walton et al., 2020). In addition to broadening the PAM tropism, much effort has been put into engineering Cas9 variants characterized by increased targeting specificity (Mueller et al., 2018). The main improvements on that end have been achieved in the studies of (Kleinstiver et al.; Slaymaker et al.; Chen et al.; and Kulcsár et al.) (Kleinstiver et al., 2016; Slaymaker et al., 2016; Chen et al., 2017; Kulcsar et al., 2017). Using an alternative approach, Kocak and colleagues also achieved higher efficiency of CRISPR/Cas9 system, by modifying the secondary structure of the gRNA spacer region in such a way that it elevates the thermodynamic barrier to gRNA binding at off-target sites (Kocak et al., 2019).

### Epigenome-editing ability and the use of CRISPR/Cas systems

**FIGURE 3:**
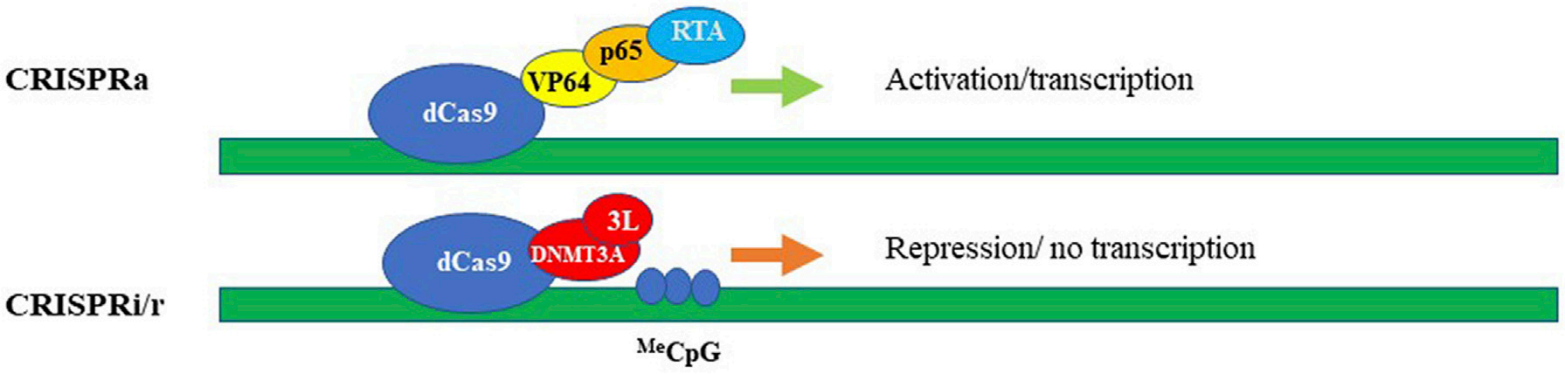
CRISPRa (activation) and CRISPRi/r (interference/repression). The synthetic activators (upper panel) comprise of dead/inactive Cas9 (dCas9) fused with transcriptional activators, e.g. VP64, p65, and RTA encoded by Epstein-Barr virus (EBV). Transcriptional repression (lower panel) schematically represented by dCas protein fused with DNA methyltransferase, here, DNMT3A and DNMT3L. The methylated DNA could be then repressed *via* the cascade of DNA-methylation mediated events.

Repurposing CRISPR/Cas systems from gene “editing” *via* the formation of DSBs to target-specific transcriptional regulation of genes became possible by modifying Cas9 to be a DNA recognition protein rather than an active nuclease (Larson et al., 2013; Qi et al., 2021), (Gilbert et al., 2013). The changes are to residues located in the two catalytic nuclease domains of the Cas9 enzyme, the RuvC domain (aspartate-to-alanine at amino acid position 10), and the HNH domain (histidine-to-alanine at position 840). These mutations abolish the endonuclease enzymatic activity of Cas9 while leaving its RNA-guided DNA targeting capacity intact (Larson et al., 2013; Qi et al., 2021; Gilbert et al., 2013). Then, the fusion of this deactivated or “dead” Cas9 (dCas9) with diverse effectors such as transcription repressors/activators, epigenetic modifiers, and others created an effective suite of tools for epigenetic modulation of gene expression (reviewed (Rittiner et al., 2020)). The modularity of dCas9 is exemplified by CRISPR interference (CRISPRi) platforms which aim to repress transcription by sterically hindering the RNA pol II machinery (Qi et al., 2013) and Figure 3B. The repression efficiency of the system has been further improved by tethering dCas9 protein to transcription repressor domains, such as the Krϋppel-associated box (KRAB) (Gilbert et al., 2013), which is present found in many zinc-finger repressors. For improved repressive efficiency, Yeo and colleagues further linked dCas9-KRAB to methyl-CpG-binding protein (MeCp2) (Yeo et al., 2018). The versatility of the dCas9–KRAB-MeCP2 system has been demonstrated by its ability to repress transcription by targeting both genes and gene-regulatory regions (Yeo et al., 2018). Nevertheless, it has to be noted that the dCas9-KRAB-MeCP2 system developed by Yeo and colleagues can fit only in plasmids or large-capacity viral delivery systems such as lentiviral vectors and adenoviruses. Smaller viruses such as AAV are currently not compatible with the bulky size of the CRISPR/Cas9 repressor platforms. Repurposing of CRISPR/Cas system also led to the development of CRISPR activator modules (CRISPRa). The first generation of these systems contain dCas9 fused either to the transcription activation domain of the NF-κB transactivating subunit (p65) or to VP64, which consists of four repeats of the herpes simplex VP16 transcription activation domain (Gilbert et al., 2013), (Perez-Pinera et al., 2013), (Maeder et al., 2013), (Farzadfard et al., 2013) and (Figure 3A). The second generation of targeted transcriptional activators based on CRISPR/Cas has been achieved by engineering gRNA-carried activators and different activator domains (Cheng et al., 2013), (Tanenbaum et al., 2014), (Konermann et al., 2015) and (Figure 3A). We will focus most of our discussion here on CRISPR/Cas-based repressive systems, as they are most relevant in aiming to develop therapeutics for neurodegenerative diseases. However, comprehensive overviews on CRISPRa systems could be found in (Rittiner et al., 2020; Anton et al., 2018; Pickar-Oliver and Gersbach, 2019). One of the first CRISPR/Cas9 systems repurposed for epigenetic silencing incorporates DNA methylation-dependent repression (Figure 3B). In fact, we and others reported robust and specific targeting using DNA methylation effectors for transcriptional deactivation by fusing dCas9 to the *de novo* DNA methyltransferase enzyme DNMT3A (Liu et al., 2016; McDonald et al., 2016; Vojta et al., 2016; Kantor et al., 2018; Tagliafierro et al., 2019). More recently, the repressive capacity of dCas9-DNMT3A systems has been further improved by tethering the former to its partner DNMT3L ((Saunderson et al., 2017) and Figure 3B), or by multiplexing the repressive signal using the “SunTag” amplification system (Huang et al., 2017). Here, to achieve signal magnification, dCas9 was conjugated to a repeating peptide epitope, which then recruits multiple copies of an antibody-effector fusion protein to the targeted genomic loci. This system tends to be less prone to non-specific editing, as has been demonstrated by Pflueger and colleagues. Indeed, they found that the utility of SunTag-DNMT3A system has resulted in a significant decrease in off-target methylation, compared to the direct dCas-DNMT3A counterpart (Pflueger et al., 2018). Our group recently developed a CRISPR/Cas9-DNMT3A system, contained in an all-in-one lentivirus, for targeted DNA methylation within a regulatory region in SNCA intron 1 (Kantor et al., 2018). Elevated levels of α-synuclein encoded by the SNCA gene have been widely implicated in the pathogenesis of Parkinson’s disease (PD), and as such we thought that targeting SNCA expression levels could be an attractive neuroprotective strategy (Tagliafierro and Chiba-Falek, 2016). In fact, in this work, we demonstrated that manipulations of SNCA expression has clear beneficial effects when delivered to human induced pluripotent stem cell (hiPSC)-derived dopaminergic neurons from PD patients carrying SNCA triplications (Kantor et al., 2018). We showed effective and specific reduction of SNCA mRNA and protein levels (Kantor et al., 2018). Importantly, this reduction was found to be coincidental to the rescue of PD-related cellular phenotypes, including mitochondrial ROS production and cellular viability in the PD derived neurons (Kantor et al., 2018), such providing a proof-of-concept validation of the developed approach as a novel epigenetics-based therapeutic strategy for PD. In addition to the use of DNA methyltransferases and general transcription factors such as KRAB to silence a gene-of-interest, the toolbox of the repurposed CRISPR/Cas systems have been further expanded to include chromatin modifiers and remodelers (reviewed in (Rittiner et al., 2020) and (Pickar-Oliver and Gersbach, 2019)). Hilton et al. demonstrated that a fusion of dCas9 and the catalytic domain of the p300 histone acetyltransferase (HAT) may cause sustained and efficient, target-specific gene activation *via* histone acetylation (Hilton et al., 2015). To achieve specific and long-term lasting repression, Kwon and colleagues fused dCas9 to histone deacetylase 3 (HDAC3) protein and showed that the developed module is capable of generating a target-specific histone deacetylation in cells (Kwon et al., 2017). To further develop this approach, Kearns et al. engineered dCas-LSD1 fusion system with the idea to directly demethylate histone H3K4. It has been demonstrated that this combination was sufficient to cause targeted loss of H3K4 methylation, which caused gene repression within the targeted loci (Kearns et al., 2015).

### Viral vector systems for delivery of CRISPR/Cas components

Virus-mediated gene therapy has traditionally been viewed as a viable long-term strategy for the disease-modifying treatment of many hereditary conditions including neurodegenerative diseases. Historically, “gene therapy” has entailed transduction using a viral vector carrying a gene- or a cDNA-of-interest aimed to compensate for a malfunctioning counterpart, or supply a different capacity to cells which enables them to be more efficient in rescuing from the disease state. Combining this ability with highly innovative CRISPR/Cas tools provides new and very appealing prospects for combating CNS diseases and disorders. Indeed, viruses are highly efficient in transducing cells and tissues, which has fascinated many investigators since the 70s (Friedmann, 1976). Commonly, vectors are depleted of all pathogenic elements, which are replaced by a transgene-of-interest. To further enhance safety of viral vectors, the various portions of their genomes are delivered into the transfected producer cells separately, from three or four plasmids. Typically, the vector plasmid contains all cis-acting elements required for efficient packaging, such as packaging signals and LTRs (lentiviral vectors) or ITRs (AAVs) (see below), in addition to the transgene. Other plasmids supply the additional components required for efficient packaging of the vector-carried genome into viral particles. As of 2019, over 3,000 clinical trials have been conducted worldwide using viral vectors (∼2% delivering therapeutic cargoes to target neurodegenerative diseases) (Corrigendum, 2019). Simple retroviral vectors (γ-retroviruses) were the first to be used in a clinical trial, aimed to correct a severe combined immunodeficiency disorder (SCID) in 1995 (Blaese et al., 1995). Tragically, it has been reported that the virus triggered severe T-cell leukemia in three children several years later. The cause of that complication was associated with the insertion of the retroviral genome into the proto-oncogene LMO2, which triggered overexpression of the corresponding protein (reviewed in Kantor et al., 2014 (Kantor et al., 2014b)). Another handicap of γ-retroviral vectors is their inability to transduce non-dividing cells, which severely limits the utility of retroviruses in the CNS. In summary, γ-retroviral vectors are not efficient for gene therapy of neurological diseases.

**FIGURE 4:**
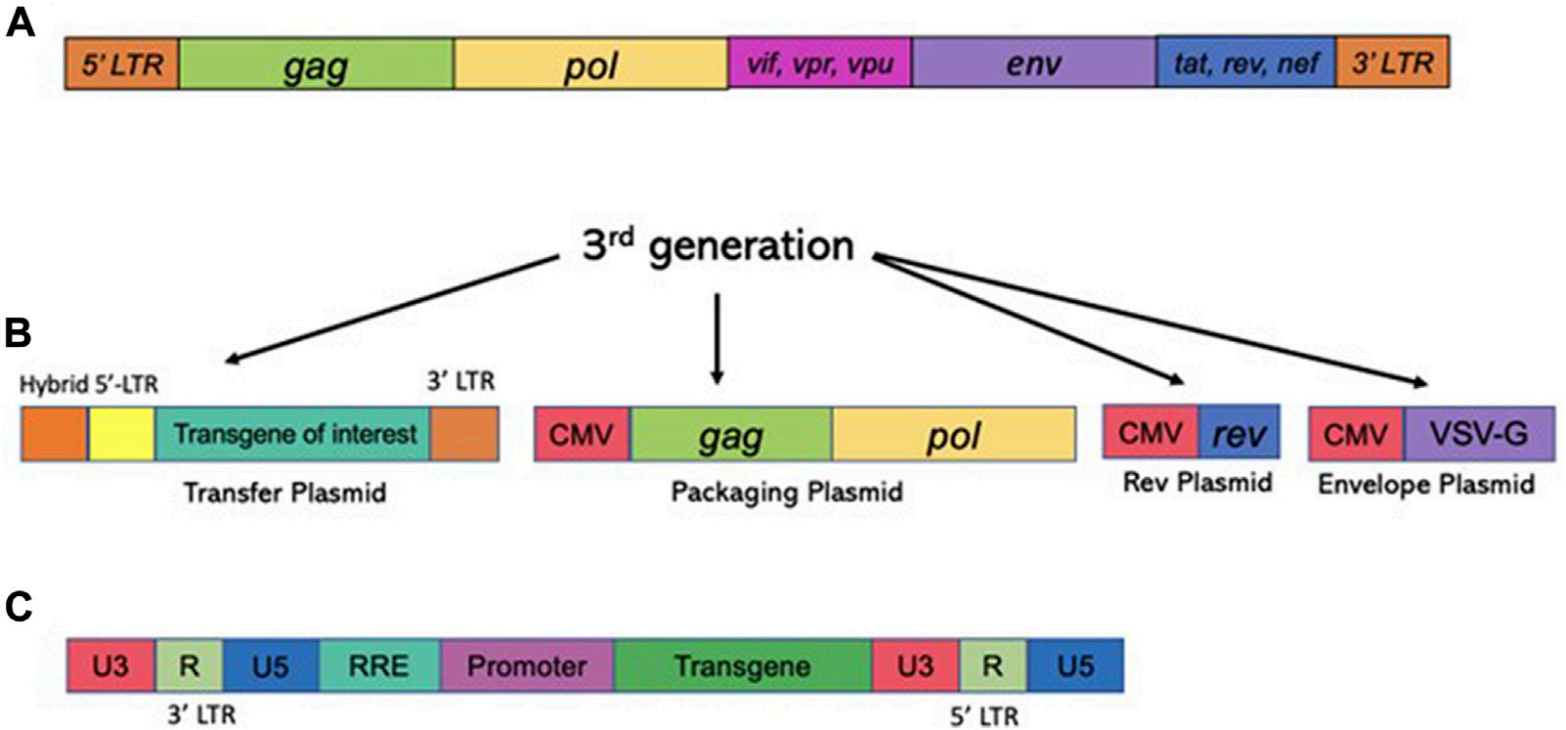
Structure of the HIV-1 based vectors (lentiviral vectors) and the packaging cassettes. The third generation of the packaging cassette is shown. Upper panel **(A)** The wild type HIV-1 genome is shown. The genome contains four accessory proteins, Vpu, Vpr, Vif, and Nef and the regulatory proteins, tat and rev. RRE stands for rev response element. The 3^rd^ generation of the packaging vector cassette is shown. (lower panel) **(B)** Expression of lentiviral vector is driven from the CMV promoter; PolyA signal (pA) is shown. The 3rd generation excluded all four accessory proteins, Vpu, Vpr, Vif, and Nef, but included regulatory proteins, tat and rev. The vector packaging cassette contains RRE and pA signals. CMV, cytomegalovirus; RRE, Rev response element. Here, the transfer cassette depicted as RNA; 5′LTR and 3′LTR are listed. **(C)** Schematic image of viral DNA following the completion of RT process. U3′ region bears the promoter which drives an expression of the transcript. Self-inactivated vectors harbor deletion in U3 region, such that the full transcript expression is not supported. The internal promoter, painted in lilac color drives an expression of a transgene-of-interest (painted in green).

Unlike γ-retroviruses, lentiviral vectors [derived from the retroviridae family, and exemplified by human immunodeficiency virus type-1 (HIV-1)] are proficient in transducing postmitotic cells, as they evolved the capability for efficient nuclear import (Lewis and Emerman, 1994). In fact, lentiviral vectors show high rates of transduction into non-dividing cells, including post-mitotic neurons *in vitro* and *in vivo* (Naldini et al., 1996). Since that work, thousands of studies have probed the use of lentiviral vectors for gene delivery into the CNS (reviewed in (Kantor et al., 2014a), (Rittiner et al., 2020)). Lentiviral vectors are an attractive delivery vehicle for the CNS, as they are capable of transducing most cells of the brain, including mitotic and postmitotic neurons, astrocytes, and oligodendrocytes (Blomer et al., 1997), (Consiglio et al., 2001), The vectors transduced into the CNS are long-lasting and are capable of robust and sustained expression (Bayer et al., 2008), (Kantor et al., 2011). An extensive coverage of lentiviral vector biology can be found in the following reviews: (Kantor et al., 2014b), (Kantor et al., 2014a), (Rittiner et al., 2020). Briefly, the lentiviral genome consists of ∼10.6 kbps of positive-sense single-stranded RNA (ssRNA), of which two identical copies are packaged inside a lipid-enriched viral capsid that is ∼100 nm in diameter. Roughly, the packaging capacity of lentiviral vectors is 10 kbps. As mentioned above, the packaging components required to form viral particles are supplied in *trans* from separate plasmids. The packaging plasmid carries the structural and enzymatic genes (*gag* and pol, respectively) but omits the genes which encode accessory proteins such as *nef, vif*, *vpr,* and *vpu,* which are dispensable for the production of viral particles (reviewed in (Kantor et al., 2014a) and Figure 4). The gag (group-specific antigen) gene produces the viral matrix (MA), capsid (CA), and nucleoproteins (NC) proteins. The *pol* ORF encodes for reverse transcriptase (RT), protease (PR), and integrase (IN) enzymes (Figure 4). Lentivirus is enveloped vector, which means that the virus needs to be equipped with a surface protein in order to be transduction-competent. Fortunately, the envelope of LV can be supplied from a heterologous source, as the modular nature of the vector supports pseudotyping process. In fact, devising different virus pseudotypes is seen as one of the major steps in adjusting tropism of the virus to broad range of cells and tissues. The constructions of various vector envelops is comprehensively addressed in (Cronin et al., 2005). Briefly, lentivirus can be efficiently pseudotyped with a wide variety of surface proteins; a portion of them, including Rabies virus (RV), Mokola virus (MV), and Ross River virus (RRV) demonstrate strong tropism towards CNS cells. With that said, typical supplementation of the vectors involves packaging with vesicular stomatitis virus protein G (VSV-G) due to its broad tropism (Cronin et al., 2005). As mentioned above, four accessory proteins encoded by HIV-1 are not necessary for the production of recombinant vector (Figure 4). Nevertheless, two regulatory proteins, rev and tat are involved in viral entry, replication, transcription, and particle release (Coffin et al., 1997). As such, the second generation of the packaging system has deleted of all accessory proteins, but still carries the tat and rev genes (Zufferey et al., 1997). The tat protein serves as a trans-activator for HIV-1 transcription. The replacement of the endogenous HIV-1 promoter located in the U3’ region of the 5′ LTR with a heterologous promoter such as Rous sarcoma virus (RSV) or cytomegalovirus (CMV) allows tat-independent transcription. As such, tat is deleted in third-generation packaging system ((Zufferey et al., 1997) and Figure 4). This packaging system is also characterized by the split of the gag/pol and rev sequences into two different plasmids, and is the safest to date (Dull et al., 1998). Both second and third generation packaging cassettes contain a strong heterologous polyadenylation signal (poly-A) from either SV40 or bovine/human growth hormone (bGH/hGH) (Dull et al., 1998), (Ortinski et al., 2017). In addition, integration of a woodchuck hepatitis virus posttranscriptional regulatory element (WPRE) and a central polypurine tract (cPPT) into the vector cassette enhances RNA stability, transcription levels, and the overall viral titer (Zufferey et al., 1999), (Zennou et al., 2000). Altogether these improvements have greatly improved the safety of lentiviral vectors by vastly reducing the probability of the formation of recombination-competent retroviruses (RCR).

The lentiviral life cycle is comprehensively covered in (Kantor et al., 2014a). Briefly, LVs enter the cells via a specific receptor-ligand interaction (reviewed in (Poeschla, 2008)). Following this process, the virus undergoes an uncoating process to expose its genetic and enzymatic loads. This step is followed by reverse transcription (RT); this reaction results in the synthesis of a double-stranded linear form of the viral DNA. This dsDNA can then be translocated into the cell nuclei where it serves as a precursor for all major DNA forms of the virus, including integrated and episomal ones (reviewed in (Rausch and Le Grice, 2004)). It worth noting that only a small portion of dsDNA integrates into the host chromosomes; the majority of viral DNA either undergoes circularization or is left linear, to form single and double LTR circles or episomal linear genomes, respectively (Kantor et al., 2009), (Bayer et al., 2008), (Kantor et al., 2011). With the conclusion of viral integration, the LV-genomes become an integral part of the host genome, as such a virus-transgene could be passed on to the cell’s progeny (Colicelli and Goff, 1985), (Bushman and Craigie, 1990), (Bushman et al., 1990), (Leavitt et al., 1992). Importantly, being part of the host genome qualifies viral mRNA to be transcribed by the host RNA polymerase II- dependent machinery.

### Risk of insertional mutagenesis: Development of integrase-deficient lentiviral vectors (IDLVs)

As mentioned above, the Achilles heel of lentivirus is rooted within its integration nature. In fact, the use of the gamma-retroviruses for clinical gene delivery is hindered by relatively high risk of insertional mutagenesis they display. For instance, positive outcomes of the treatment of ADA-SCID, SCID-X1, and X-linked CGD diseases with gamma-retroviral vectors have been unfortunately overshadowed by blood cancers induced in several patients. As mentioned above, that was due to the integration of retroviral cassettes carrying the therapeutic transgene in the vicinity of proto-oncogenes (Cavazzana-Calvo et al., 2000; Hacein-Bey-Abina et al., 2003a; Hacein-Bey-Abina et al., 2003b). A high-risk of mutagenesis is characteristic to all members of retroviral family of viruses including LVs. As mentioned above, LVs are integrating viruses by default; as such, one could theoretically retain the ability to trigger onco- and tumorigenicity. Albeit, there is no convincing evidence to support this notion so far, lentiviral vectors based on equine infectious anemia virus (EIAVs) have been linked to the formation of tumors in the livers of mice following in utero- and neonatal vector administration (Themis et al., 2005). Again, it must be noted that a causal relationship between EIAVs and cancer has yet to be established. Indeed, the results reported in the same study report that there were no tumors observed upon the use of human LVs (Themis et al., 2005). Nevertheless, the development of safer LV systems would be highly desirable for the field of gene therapy. A strategy to lower the integration capacity of LVs is based on developing an episomal vector platform. We and others have previously demonstrated that episomal forms of HIV-1 and LV constitute the vast majority of viral genomes (Kantor et al., 2011; Chun et al., 1997). Furthermore, we reported that those episomal forms are stable in nondividing cells (Kantor et al., 2011). Characterization of episomal forms led to the discovery of four major DNA forms: linear (lDNA), 2-LTR (double-LTR), 1-LTR (single-LTR), and some other aberrant circular forms. All those forms are the product of host-mediated repair activity related to homologous and non-homologous recombination (Bayer et al., 2008; Kantor et al., 2011). Production of integrase-deficient (IDLV) lentiviral vectors is based on the identification of nonpleiotropic mutations in the catalytic motif of the integrase enzyme (Engelman et al., 1995; Nakajima et al., 2001). A point mutation in this motif can completely abolish the integration process without affecting any of other steps in the virion formation. We demonstrated that IDLVs are capable of expressing therapeutic cargoes, albeit at levels lower than those seen with integrase-competent LVs (Kantor et al., 2009; Bayer et al., 2008; Kantor et al., 2011). We were recently able to significantly upgrade IDLVs *via* changes introduced into viral expression cassette (Ortinski et al., 2017; Vijayraghavan and Kantor, 2017; Tagliafierro et al., 2019). The optimized system was shown to be very efficient. The above study suggests that integrase-deficient lentiviral vectors could be a safe, efficient, useful viral platform for a broad range of gene therapy applications. The CRISPR/Cas9 systems have revolutionized the field of genome editing by providing unprecedented control over gene sequences and gene expression in many species, including humans. Lentiviral vectors are one of the primary delivery platforms for CRISPR/Cas9 systems due to their ability to accommodate large DNA payloads and sustain robust expression in a wide range of dividing and non-dividing cells. However, long-term expression of LV-delivered Cas9/guide RNA may lead to undesirable off-target effects characterized by non-specific RNA-DNA interactions and off-target DNA cleavages. Integrase-deficient lentiviral vectors (IDLVs) present an attractive means for delivery of CRISPR/Cas9 components because: 1) they are capable of transducing a broad range of cells and tissues, 2) they have superior packaging capacity compared to other types of vector (e.g., adeno-associated viral vectors), and 3) they are expressed transiently and demonstrate very weak integration capability. As such, we aimed to establish IDLVs as a means for safe and efficient delivery of CRISPR/Cas9. To this end, we developed an all-in-one vector cassette with increased production efficacy and demonstrated that CRISPR/Cas9 delivered by the improved IDLV vectors can mediate rapid and robust gene editing both in human embryonic kidney (HEK293T) cells *in vitro* and post-mitotic brain neurons *in vivo* (Ortinski et al., 2017). In addition, we demonstrated that the addition of the transcriptional enhancer binding sites into all-in-one vector cassettes results in a significant increase in the packaging efficiency of the IDLV vectors. Notably, the optimized integrase-deficient vectors have shown improved specificity over their integrase-wt counterparts, as they induced substantially lesser levels of off-target effects (Ortinski et al., 2017). More recently, we demonstrated that the optimized vectors carrying an epigenome-editing transgene based on the CRISPR interference (CRISPRi) system were capable of functionally decreasing overexpressed alpha-synuclein protein by reducing transcription levels of SNCA gene (Kantor et al., 2018). In fact, applying the system to human induced pluripotent stem cell (hiPSC)-derived dopaminergic neurons from a Parkinson’s disease (PD) patient with a SNCA triplication resulted in downregulation of both SNCA mRNA and α-Syn protein, mediated by targeted DNA methylation at intron 1 (Kantor et al., 2018). Furthermore, the reduction in SNCA levels by the guide RNA (gRNA)-dCas9-DMNT3A (*de novo* methyltransferase 3A) system rescued disease-related cellular phenotype characteristics of the SNCA triplication hiPSC-derived dopaminergic neurons, e.g., mitochondrial ROS production and cellular viability (Kantor et al., 2018). The novel system has been validated *in vivo*, and has displayed successful downregulation of the endogenous mouse SNCA gene, as well as the human counterpart overexpressed from a separate transgene virally delivered into the mice brain (personal communication, 2022). These experiments suggest that the novel epigenetic-based therapeutic approach developed in our laboratory for treatment of PD and other hereditary diseases has high translational value (Rittiner et al., 2020).

### Adeno-associated vectors (AAVs) for applications involving CRISPR/Cas delivery

**FIGURE 5:**
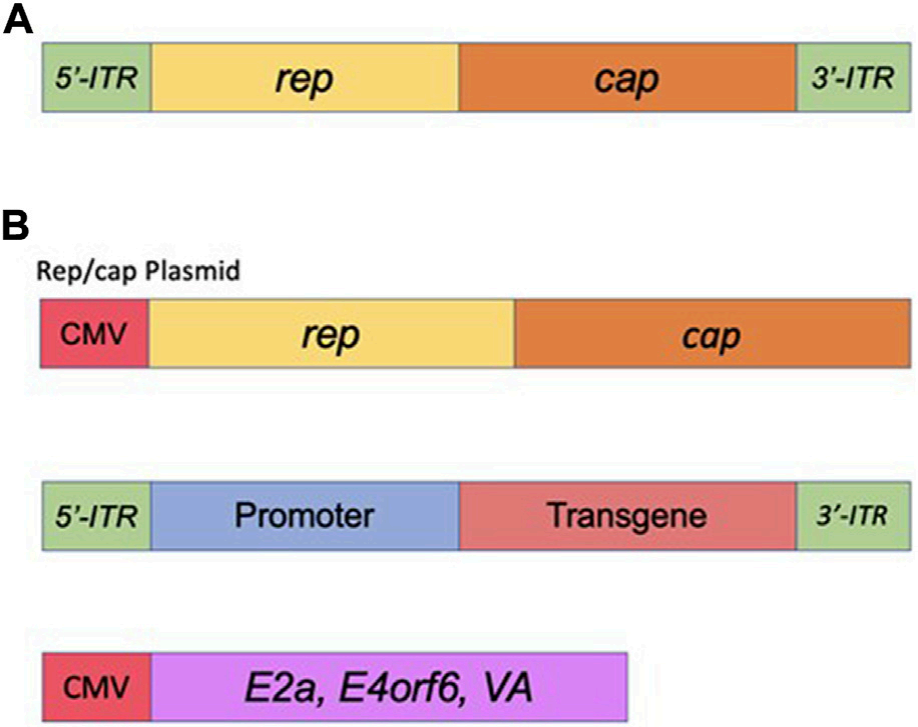
Adeno-Associated Virus (AAV) basics. **(A)** Simplified schematic of the wild-type AAV genome. **(B)** Plasmids used in the current AAV packaging system. See the main text for a detailed description of the AAV packaging system.

Adeno-associated viral vectors (AAVs) are the platform-of-choice for the delivery of therapeutic genes (Kantor et al., 2014a). The recombinant AAV (rAAV) variants have been cloned from the wt AAV virus—a member of the Dependovirus genus. As with other members of the genus, AAV completely depends on coinfection with a helper virus such as adenovirus or HSV to sustain its replication cycle in the host cells (reviewed in (Kantor et al., 2014b)). The genome of wt AAV consists of 4.7 kb of ssDNA and is structurally quite simple: two ORFs, *rep* and *cap*, are flanked by a pair of 145 bp inverted terminal repeats (ITRs) ((Lusby et al., 1980), (Srivastava et al., 1983), (Sonntag et al., 2010) and Figure 5). A total of eight proteins are produced from this genome; this includes four proteins from *rep* (Rep78/68 and Rep52/40), and three capsid proteins (VP1, VP2, and VP3) plus an assembly activating protein (AAP), all encoded by *cap* (reviewed in (Kantor et al., 2014a) and Figure 5). The two larger Rep proteins mediate viral replication and integration, and the shorter variants are involved in the packaging of the viral DNA into viral particles (reviewed in (Kantor et al., 2014a)). The structural VP1, VP2, and VP3 capsid proteins are produced at a ratio of 1:1:10, respectively (Kronenberg et al., 2001); sixty total copies of these proteins (at the same ratio) combine to form each icosahedral AAV virion. Lastly, the above mentioned AAP protein is involved in trafficking capsid proteins to the nucleolus (the site of virion assembly) and is also important in the process of capsid formation and assembly (Smith, 2008). As mentioned above, AAV system represents the current platform-of-choice for viral-mediated gene-to-cell transfer. First, both recombinant AAVs and the wild-type virus are safe; they are not associated with any known pathology or disease. Second, similar to the IDLV delivery system, AAVs are episomal vectors incapable of integration into the host chromatin. Thus, the episomes will persist over the long term in non-dividing and slowly-dividing cells, but would be quickly diluted out by cell division in dividing cells. Long-term expression of the AAV genome in non-dividing cells is well-characterized (reviewed in (Kantor et al., 2014b)). The virus supports a broad range of tropisms to various cells and tissues, and many novel capsids have recently been engineered through various methods of directed evolution of the vector. Importantly, some of the new capsids are significantly less immunogenic compared with the natural serotypes (Wu et al., 2006). Last but not least, AAV structural organization is well-understood, so the outcomes of genome manipulations can be reasonably be projected. AAV vectors have undergone a great deal of optimizations and improvements since rAAV was first established about 40 years ago (Samulski et al., 1983). That long journey has brought AAV to be a gold-standard and transformative platform for gene therapy. Here, we will briefly outline the main milestones of rAAV transformation. It was first demonstrated that the stem-loop-forming inverted terminal repeats (ITRs) are the only cis-acting elements required for production of recombinant virus (Lusby et al., 1980), (Nash et al., 2008). That important discovery led to the design of a packaging plasmid which provides the rep and cap genes in *trans*. Importantly, the split of the packaging and the expression cassette into two plasmids allowed for the room to insert a transgene-of-interest. Still, the main bottleneck of AAV is that it can only carry relatively small transgenes, whose size does not exceed 4.7 kb (including the ITRs). Another advantage of splitting packaging system is that it prevents the possibility of recombining viral genome into wild-type AAV during the production phase (Kantor et al., 2014a). Furthermore, with no inclusion of the *rep* gene the above separation minimizes integration capacity of AAV, as opposed to the wt virus, which is capable of integrating into human chromosome 19 (Young et al., 2000). In fact, rAAV appears to integrate randomly at a low rate (integration occurs in 0.1–1% of the transduced cells), with the vast majority of DNA being maintained as episomes (Kantor et al., 2014a). The next major step in optimization of AAV production was the substitution of the helper virus with the required proteins in *trans*. As mentioned above, helper function required for AAV production originally was supplied by co-infecting the cells with Adenovirus or HSV-1. Needless to say that this method results in high levels of contamination of the preps with the helper viruses. To circumvent this issue, Xiao and colleagues designed a plasmid expressing only the essential adenovirus helper genes: E1a, E1b, E2a, E4orf6, and viral-associated RNA genes (Xiao et al., 1998) (see also, Figure 5). Notably, human embryonic kidney cells, HEK293T, utilized for the production, carry E1a and E1b genes, eliminating the need in their complementation from the helper cassette (Xiao et al., 1998). The above improvements have supported a mass production protocol for recombinant virus, characterized by its diminished immunogenicity leading to the broad use of AAV for a variety of gene transfer applications, including human gene therapy based on the delivery of CRISPR/Cas transgenes. Nevertheless, despite the impressive and rapidly diversifying array of CRISPR/Cas-derived tools, the packaging limit of AAV genomes is still the main hurdle, especially when the high-titer virus is desired. To circumvent the significant restraints imposed by AAV’s ∼4.7 kb functional packaging capacity, several groups have devised the following approach. A bulky and multi-component CRISPR/Cas transgene is physically split into two pieces, which are packaged into separate AAVs. The resulting AAVs are then co-delivered, and the complete protein is reassembled *in situ* by a split-intein—a pair of domains which “splice themselves out,” thus joining two peptide chains end-to-end (Chew et al., 2016), (Moreno et al., 2020). Still, the advantage of all-in-one systems which provide higher-level of efficacy clearly justifies the use of IDLV systems capable of carrying large/multicomponent transgenes. Integrase-deficient vectors may prove critical for the delivery of next generations of CRISPR/Cas tools, including prime- and base-editors, as well as epigenome-editing tools (highlighted below), as the complete systems with all the included elements would not fit even in a dual-AAV delivery format. One major focus of the innovative gene-editing technology in which viral-mediated delivery systems (AAV, LV and IDLV) are commonly utilized, has been *ex vivo* engineering of cellular therapies. The process involves collection of specific cells from a patient, which are then gene-edited and engrafted. In such way, for example, CD4^+^ T cells harvested from HIV-positive patients have been edited to delete the CCR5 locus and thereby confer resistance to re-infection, followed by re-engrafting into the same patients (Xu et al., 2017; Lin et al., 2021; Hutter et al., 2015). The donor template for the above editing is commonly provided *via* AAV or IDLV delivery. Another example for *ex-vivo* use of AAV or IDLV systems for the delivery of donor sequences engaged in the HDR-mediated DNA repair is the reprogramming T cells to mediate tumor rejection. To that end, chimeric antigen receptors (CARs) are used. The most successful CARs used to date are those targeting CD19, which offer the prospect of complete remission in patients with chemorefractory or relapsed B-cell malignancies (reviewed in (Mollanoori et al., 2018)). For *in vivo* editing, AAV’s packaging capacity posed initial challenges for CRISPR/Cas9 delivery, as the combined size of the initially best-characterized *Streptococcus* pyogenes Cas9 (SpCas9), the sgRNA, and promoters for each was simply too large to fit into a single AAV vector. However, two primary approaches for utilizing AAV as a CRISPR/Cas9 delivery vector have since emerged. Since the initial discovery and characterization of SpCas9, thousands of CRISPR/Cas9 proteins have been identified (Poon et al., 2006), many of which are significantly smaller than SpCas9. The best-characterized alternative Cas9 protein, derived from *Staphylococcus aureus* (SaCas9), is nearly 1 kb shorter than SpCas9 and can thus be accommodated along with its sgRNA in AAV (Machida et al., 2018). Other non-Cas9 CRISPR proteins, such as Cpf1 (Tachibana et al., 2005), offer new binding and cleavage characteristics in addition to being more compact. With these smaller CRISPR/Cas9 proteins, the entire system can finally fit in a single AAV vector. As an alternative, some studies have packaged SpCas9 and the sgRNA in separate vectors for co-administration (Peters et al., 2003). This approach is particularly useful for HDR-modification applications, where, for example, one vector could be used to deliver the nuclease and sgRNA and a second vector the HDR template.

## Discussion

In the discussion session, we would like to extend on some of the most recent developments in the field as well as address some of shortcomings of the CRISPR/Cas systems.

### CRISPR/Cas-based therapies paired with AAV delivery

Both the use of smaller Cas9s and the dual vector approach have been successfully implemented *in vivo* for an increasing number of applications, both to disrupt endogenous gene expression as well as to precisely correct disease alleles. In early 2015, SaCas9 and its sgRNA were combined in a single AAV8 vector to disrupt and thereby knock out expression of a cholesterol regulatory gene, proprotein convertase subtilisin/kinexin type 9 (PCSK9), in the adult mouse liver (Machida et al., 2018). The result was reduced circulating cholesterol levels. Furthermore, an “all-in-one” AAV has been generated to deliver both SaCas9-and sgRNA-machineries, along with a self-linearizing repair template. The vector was found to be efficient for correcting Fah mutation in mice to treat HT-1 (Krooss et al., 2020). Recently, investigators profiled Type VI CRISPR/Cas systems to engineer a Cas13 ortholog capable of robust target-specific knockdown of RNA (Cox et al., 2017). The authors demonstrated that RNA editing *via* catalytically-inactive Cas13 (dCas13) could be efficient to direct adenosine to inosine deaminase activity by ADAR2 to transcripts in mammalian cells. The system, coined as RNA Editing for Programmable A to I Replacement (REPAIR), has shown no strict sequence constraints, and could be exploit to edit full-length transcripts containing pathogenic mutations (Cox et al., 2017). Importantly, Cox and colleagues demonstrated that the next generation of the above system, carrying an ADAR2DD (E488Q) variant was small enough to fit within the packaging limit of AAV vectors and was able to efficiently edit targeted RNA *in vivo* (Cox et al., 2017). In a different study, Konermann and colleagues analyzed prokaryotic genome and metagenome sequences to identify an uncharacterized family of RNA-guided, RNA-targeting CRISPR systems which was classify as Type VI-D (Konermann et al., 2018). The Type VI CRISPR-Cas13 superfamily is exemplified by sequence-divergent, single-effector signature nucleases and the presence of two HEPN domains (an ortholog of the Ruv endonuclease/recognition domains found in Cas9). Biochemical characterization and protein engineering of Cas13 has yielded robust activity in human cells. The Cas13d family of proteins averages 930 amino acids in length, in contrast to Cas9 (∼1100–1400 aa depending on subtype, with compact outliers such as CjCas9 or SaCas9), Cas13a (1250 aa), Cas13b (1150 aa), and Cas13c (1120 aa). The remarkably small size of Cas13d effectors render them uniquely suited for all-in-one AAV delivery with a CRISPR array, an optional effector domain, and promoter and regulatory elements. Konermann and colleagues assembled an all-in-one AAV vector of the above CRISPR system to treat Frontotemporal Dementia with Parkinsonism linked to Chromosome 17 (FTDP-17)—an autosomal dominant major neurodegenerative disease caused by diverse point mutations in MAPT, the gene encoding for the tau protein. Specifically, the authors targeted FTD which stemmed from mutations in the intron following MAPT exon 10. The mutation has been shown to disrupt an intronic splice silencer and elevate the expression of 4R tau, disturbing the balance between 4R and 3R, which are distinguished by the presence or absence of tau exon 10 and thus contain 4 or 3 microtubule binding domains. The balance between two isoforms seems to be crucial, as it is generally perturbed in FTDP-17 as well as other tauopathies, driving the progression of neurodegeneration (Boeve and Hutton, 2008). The authors reasoned that the novel system targeted to MAPT exon 10 could induce exon exclusion to alleviate dysregulated 4R/3R tau ratios. As such, they applied it to patient-derived human induced pluripotent stem cells (hiPSCs) which had been differentiated into cortical neurons. These neurons were then transduced with AAV1 carrying all in one AAV vector paired with a repeat array containing 3 spacers that target the exon 10 splice acceptor and two putative exonic splice enhancers (Konermann et al., 2018). dCas13-mediated exon exclusion was able to reduce the relative 4R/3R tau ratio by nearly 50% relative to a BFP vehicle control, and to a level similar to unaffected control neurons, suggesting that the novel all-in-one system can be exploited for transcriptional modulation in primary cell types *via* AAV delivery (Konermann et al., 2018). It is unclear at this point if the challenges associated with “classic” Cas9 vectors would apply to the novel and engineered Cas9 proteins. For example, pre-existing humoral and cellular immunity against commonly used Cas9 orthologs has been reported in general human populations due to widespread infections of the bacteria from which these proteins are derived (Crudele and Chamberlain, 2018; Wagner et al., 2019). It remains to be evaluated whether delivering vectored Cas9 proteins directly into the human body in the presence of pre-existing anti-Cas9 immunity will compromise safety or therapeutic efficacy. Potentially, various protein engineering approaches can improve clinically relevant features, such as mapping and editing epitopes for a better immunological profile of the AAV-CRISPR systems. Along with the continuing successes in proof-of-concept animal studies such as those mentioned above, the first human therapeutic application for CRISPR/Cas has gained regulatory approval. A phase 1/2 clinical trial employing AAV-CRISPR delivery directly to the eye to correct a CEP290 mutation is currently open for LCA10 patient enrollment (ClinicalTrials.gov Identifier: NCT03872479). Along with the CEP290 mutation mentioned above, about 6% of Leber congenital amaurosis (LCA) cases are caused by mutations in RPE65 (den Hollander et al., 2008). As such, RPE65-associated retinal dystrophy is an ideal target for gene therapy. To date, the principal treatment for LCA have been developed by Spark Therapeutics. The voretigene neparvovec (Luxturna, Spark Therapeutics), was approved by the U.S. Food and Drug Administration (FDA) in December 2017. The approach is based on RPE65 gene delivery by adeno-associated virus type 2 (AAV2) to the retina in patients who lack the functional RPE65 protein (https://www.fda.gov/vaccines-blood-biologics/cellular-gene-therapy-products/luxturna). Not surprisingly, RPE65-targeting *via* CRISPR/Cas gene-editing systems have been a frontrunner for the development of a new treatment for LCA. In a recent study, Jo and colleagues have deployed AAV-CRISPR-Cas9–mediated HDR as a new treatment for LCA (Jo et al., 2019). The group demonstrated that dual AAV–mediated delivery of CRISPR-Cas9 and an Rpe65 donor sequence can lead to the correction of a disease-causing mutation in Rpe65 and an improvement in retinal function in a mouse model of LCA (Jo et al., 2019). In addition to this improvement, this study showed a level of functional recovery that exceeds the measured gene correction level. As mentioned above, the preclinical studies demonstrated successful correction of CEP290 mutation enabled IND approval for LCA10 patient enrollment. The most prevalent CEP290 mutation causing LCA10 is IVS26, which introduces a premature stop codon *via* alterations to RNA splicing (Burnight et al., 2017). In the phase 1/2 EDIT-101 trial an AAV5 vector is used to deliver SaCas9 and CEP290-specific guide RNAs to photoreceptor cells by subretinal injection. Wild-type CEP290 mRNA is produced *via* intronic inversion or deletion mediated by the editing construct. In the case of LCA10, a minimum gDNA editing efficiency of 10% was determined in earlier studies to be required for meaningful vision restoration, and this baseline efficiency was exceeded in mouse and nonhuman primate models (Maeder et al., 2019). Final results from the EDIT-101 trial for LCA10 are expected in 2024, although initial clinical data from the phase 1/2 BRILLIANCE trial (Editas Medicine, Cambridge, Mass.) showed a positive safety profile at 15 months after treatment and limited evidence of clinical efficacy. Rationally, the great progress achieved in eye-CRISPR-mediated treatments has been replicated in the liver-associated diseases. The liver is a highly favorable target for gene therapy for a number of reasons. Every minute, the liver filters 1 L of blood arriving from the portal vein and hepatic artery, and hepatic sinusoid structures allow viral vectors in the bloodstream to directly transduce hepatocytes. As a result, systemically delivered therapies (e.g. intravenous injection) result in robust expression in the liver. Furthermore, expression of transgenes in hepatocytes remains stable over time. Dilution over time is not a major concern, as cell turnover rates are low (1–2%) and the turnover process is lengthy. While AAV8 and AAV9 serotypes have particularly strong tropism towards human hepatocytes, several other serotypes do as well: AAV2 and AAV5 also show liver tropism, and several other AAV serotypes have been demonstrated in various animal models, including non-human primates. All of these serotypes have been utilized in gene therapy clinical trials targeting the liver, as well as various hybrids and engineered variants. Notable examples are current Phase 3 trials for Hemophilia A and B. Early positive results from Phase 3 trials of UniQure’s AMT-061 (atranaogene dezaparvovec), an AAV5 vector with the factor IX (FIX) transgene for Hemophilia B, and BioMarin’s BMN 270 (valoctocogene roxaparvovec), an AAV5 vector with the factor XII (F8) transgene for Hemophilia A, emerged in 2021. The clinical use of nucleases in treating liver-associated pathologies, successfully set by Sangamo Therapeutics. In fact, building upon the many successes with genome editing in animal models, the first clinical trial of genome editing therapy (NCT03041324) led by the company took place in November 2017 using AAV delivery of ZFNs. This Phase 1/2 clinical trial attempts to treat MPS II (mucopolysaccharidosis II) patients with mutations in the iduronate-2-sulfatase (IDS) gene by inserting a correct copy of the IDS gene into the albumin locus in hepatocytes. Similar clinical trials were performed for MPS I (NCT02702115), which is caused by mutations in the IDUA gene, and for hemophilia B (NCT02695160) patients, which is caused by mutations in the factor IX (FIX) gene. Unfortunately, all of the three clinical trials failed to demonstrate clinical benefit, probably due to low genome editing activities attributed to either suboptimal activity of the first-generation ZFNs, low activity of HDR mediated insertion, or insufficient delivery by the AAV vector. Detailed analysis and scientific publication are awaited. It is well known that ZFNs and other unmodified nucleases possess unwanted off-target effects, associated with the double strand break (DSB) which may be induced off target gene or sequence-of-interest. The same caveat, unfortunately, is well documented for wild type Cas enzymes. In fact, the intrinsic risk of generating DSBs, for example activation of p53 and potential chromosomal translocations, makes less “invasive” genome editing, for instance using a nickase Cas (Cas-nickase) that introduces a single-stranded DNA break, a safer option for *in vivo* gene therapy (reviewed in (Rittiner et al., 2020)). Two recent papers by Rothgangl et al. and by Musunuru et al. use the latter approach and report Cas9-nickase targeting of a DNA adenine deaminase (ABE)—an enzyme that catalyses an adenine-to-guanine conversion—to introduce a point mutation in the first splice donor site of the proprotein convertase subtilisin/kexin type 9 (PCSK9) gene specifically in hepatocytes, and with high efficiency (Rothgangl et al., 2021), (Musunuru et al., 2021). In these studies, dual AAV vector delivery systems were used to induce base editing. Nevertheless, the studies present a major step forward by demonstrating that transient expression of ABEs by AAV delivery to the liver is not only very effective in knocking out a gene but also seems to be very safe, at least in animals, both indicating this method is highly suitable for treating familial forms of hypercholesterolaemia but also potentially other severe monogenetic liver disorders. Most recently these studies progressed into clinical trials, in which first in the clinic is Verve Therapeutics’ VERVE-101, a liver-targeted PCSK9-silencing base editor developed in collaboration with Beam Therapeutics (Kingwell, 2022). The phase Ib trial in heterozygous familial hypercholesterolaemia started in New Zealand in July 2022. Verve plans to enroll around 40 patients and will present interim data next year. The company also intends to submit an investigational new drug (IND) application to the FDA later this year. The liver/cardiovascular opportunity does not stop at PCSK9, however. Verve is also working on a base editor that silences ANGPTL3, another validated lipid-lowering liver target; an ANGPTL3-specific antibody therapy, evinacumab, is approved for homozygous familial hypercholesterolaemia. Sequential dosing of PCSK9 and ANGPTL3 base editors had additive effects on lipid levels in nonhuman primates. The study has been reported at the American College of Cardiology (ACC) annual meeting in April 2022. The report by Verve Therapeutics has shown durable and well-tolerated editing of ANGPTL3 gene out to more than 20 months in non-human primates (press release, https://ir.vervetx.com/news-releases/news-release-details/verve-therapeutics-reports-durable-and-well-tolerated-editing). Remarkably, the founder of Beam Therapeutics, David Liu, and his group at the Broad Institute most recently reported AAV with size-optimized genomes incorporating compact adenine base editors (ABEs) that enable efficient editing in mice. In fact, single-AAV-encoded ABEs retro-orbitally injected in mice led to editing efficiencies in liver (66%), heart (33%) and muscle (22%) tissues that were up to 2.5-fold those of dual-AAV ABE8e, and to a 93% knockdown (on average) of human PCSK9 and of mouse Pcsk9 and Angptl3 in circulation, concomitant with substantial reductions of plasma cholesterol and triglycerides (Davis et al., 2022). Moreover, three size-minimized ABE8e variants, each compatible with single-AAV delivery, collectively offer compatibility with protospacer-adjacent motifs for editing approximately 82% of the adenines in the human genome (Davis et al., 2022). The authors suggest that ABEs encoded within single AAVs will facilitate research and therapeutic applications of base editing by simplifying AAV production and characterization, and by reducing the dose required for the desired level of editing. Sangamo Therapeutics, mentioned above, also set the stage with the development and utility of epigenome-editing tools for correcting human diseases. The company paired with Novartis and Biogen to advance its proprietary platform for the next generation of neurodevelopmental treatments. Several neurological conditions, including autism spectrum disorder (ASD) are to be targeted *via* this collaboration. The collaboration will leverage Sangamo’s propriety genome regulation technology, zinc finger protein transcription factors (ZFP-TFs), aiming to upregulate the expression of key genes involved in neurodevelopmental disorders. Novartis will contribute its proprietary AAV system developed for chimeric antigen receptor T-cells (CAR-Ts) programs media release, https://investor.sangamo.com/news-releases/news-release-details/sangamo-announces-global-collaboration-novartis-develop-genomic. Furthermore, Biogen and Sangamo recently announced a global collaboration to develop gene regulation therapies for Alzheimer’s, Parkinson’s, and other neuromuscular and neurological diseases. The companies will combine their efforts to develop ST-501 for tauopathies including Alzheimer’s disease, ST-502 for synucleinopathies including Parkinson’s disease, a third undisclosed neuromuscular disease target, and up to nine additional undisclosed neurological disease targets. On the same note, the study from the Sangamo group most recently validated the ZFN-TF system paired with AAV vectors towards reduction of the neuronal tau, which conferred resilience against β-amyloid and tau-related neurotoxicity *in vitro* and *in vivo* (Wegmann et al., 2021). In this study, Wegmann and colleagues introduced a novel translational approach to lower expression of the tau gene MAPT at the transcriptional level using gene-silencing zinc finger protein transcription factors (ZFP-TFs). The study reported that following a single administration of AAV vector, either locally into the hippocampus or intravenously to enable whole-brain transduction, resulted in selective reduction of tau mRNA and protein by 50–80% out to 11 months, the longest time point studied. Importantly, sustained tau lowering was achieved without detectable off-target effects, overt histopathological changes, or molecular alterations. Most importantly, Tau reduction with AAV ZFP-TFs was able to rescue neuronal damage around amyloid plaques in a mouse model of Alzheimer’s disease (APP/PS1 line). The highly specific, durable, and controlled knockdown of endogenous tau makes AAV-delivered ZFP-TFs a promising approach for the treatment of tau-related human brain diseases (Wegmann et al., 2021). More exciting news has recently come from Stanley Qi’s group at Stanford: the development of a compact and versatile CRISPR-Cas system that enables genome engineering applications through high-efficiency AAV-based delivery in a wide variety of contexts (Xu et al., 2021). In this study, Xu and colleagues created an efficient miniature Cas system (CasMINI) engineered from the type V-F Cas12f (Cas14) system by guide RNA and protein engineering; Cas12f is less than half the size of currently used CRISPR systems (Cas9 or Cas12a). They demonstrated that CasMINI can drive high levels of gene activation (up to thousands-fold increases), while the natural Cas12f system fails to function in mammalian cells (Xu et al., 2021). They showed that the CasMINI system has comparable activities to Cas12a for gene activation, is highly specific, and allows robust gene, base, and epigenome editing. Importantly, Xu and colleagues demonstrated that the novel CasMINI can be efficiently packaged into all-in-one AAV viral particles for cell engineering and gene therapy applications *ex vivo* and *in vivo* (Xu et al., 2021). Most recently, Qi’s company Epic Bio got funded by Horizons Ventures to develop the GEMS (Gene Expression Modulation System) platform for precise modification of gene expression programs. GEMS includes the largest known library of novel modulators combined with advanced functional and computational genomics capabilities to rapidly design guide RNAs that are highly specific to the targeted genes. Series A funding from Horizons will support Epic’s preclinical programs in five initial indications that are insufficiently addressed by today’s genetic medicines—Facioscapulohumeral Muscular Dystrophy (FSHD), Heterozygous Familial Hypercholesterolemia (HeFH), Alpha-1 Antitrypsin Deficiency (A1AD), Retinitis Pigmentosa 4 (RP4), and Retinitis Pigmentosa 11 (RP11) (https://www.businesswire.com/news/home/20220712005353/en/Epic-Bio-Founded-by-CRISPR-Pioneer-Launches-to-Revolutionize-Genetic-Medicine-With-Epigenetic-Engineering).

Finally, we recently spun out from Duke University a startup, CLAIRIgene, which aims to advance precision medicine in Alzheimer’s disease, related dementias, Parkinson’s disease, and other Lewy body spectrum disorders *via* gene therapy approaches. The company committed to bring gene-targeted epigenome therapies for unmet medical needs in CNS disorders with a focus on age-related neurodegenerative diseases. As summarized in Rittiner and colleagues’ review on the topic (Rittiner et al., 2020), the company devotes attention to lentiviral and adeno-associated viruses as efficient vehicles to deliver all-in-one CRISPR/Cas tools for epigenome-based therapies. The first generation of the CRISPR/Cas systems developed by Kantor and colleagues has been proven to be efficient and safe in targeting the regulation of SNCA expression, dysfunction of which has been shown to be the case in Parkinson’s disease (reviewed in (Tagliafierro and Chiba-Falek, 2016)). DNA methylation at SNCA intron 1 regulates SNCA transcription, and PD brains show differential methylation levels compared to controls. Thus, DNA methylation at SNCA intron 1 is an attractive target for fine-tuned downregulation of SNCA levels. Kantor and colleagues developed a system, comprising an all-in-one lentiviral vector, for targeted DNA methylation editing within intron 1. The system is based on CRISPR-deactivated Cas9 (dCas9) fused with the catalytic domain of DNA-methyltransferase 3A (DNMT3A). Applying the system to human induced pluripotent stem cell (hiPSC)-derived dopaminergic neurons from a PD patient with the SNCA triplication resulted in downregulation of SNCA mRNA and α-Syn protein, which was mediated by targeted DNA methylation at intron 1. Furthermore, the reduction in SNCA levels by the guide RNA (gRNA)-dCas9-DMNT3A system rescued disease-related cellular phenotype characteristic of the SNCA triplication in hiPSC-derived dopaminergic neurons, e.g. mitochondrial ROS production and cellular viability. Furthermore, we established that DNA hypermethylation at SNCA intron 1 allows an effective and sufficient tight downregulation of SNCA expression levels in rodent model *in vivo*, suggesting the potential of this target sequence combined with the CRISPR-dCas9 technology as a novel epigenetic-based therapeutic approach for PD. Most recently, CLAIRIgene and Seelos Therapoetics merged effort to advance the technology towards IND-enabling studies, with the expectation to move it towards clinical study in PD patients. As pointed out above, one of the most notable things about CRISPR/Cas-based gene therapy is how rapidly it has evolved. But as exciting as this development, experts in fields ranging from science, medicine to bioethics, including our group, have cautioned that the progress in the clinical applications related to the technology, should not overtake the associated shortcomings related to research and ethical complications that arise (Yang et al., 2021; Angrist et al., 2020; MacDougall et al., 2021). In fact, the ethical issues related to the CRISPR/Cas technology have made headlines most recently. Dr. He Jiankui, a Chinese biophysicist, announced in 2018 that he had engineered the first genetically altered offspring, by editing the CCR5 receptor gene in the human embryos’ germline, to make the babies less susceptible to HIV. The study has created an enormous outrage in the scientific and ethical communities, and He Jiankui was ultimately sentenced to serve three year-prison for illegal misconduct. This should be used as a reminder that the easy-to-use technologies, such as CRISPR/Cas has the high risk to be abused without clear directive and oversight from the regulatory, government entities. Furthermore, as mentioned above, relatively high risk of undesirable off-target effects still a key caveat for exploiting CRISPR/Cas technology in clinical applications. Much effort has been done on both fronts, as so we are hopeful that this revolutionary, but new technology will move forward efficiently, and safely.

## Conclusion

Using CRISPR/Cas tools paired with viral mediated gene-to-cell transfer is an attractive and novel perspective, especially when it comes to the treatment of genetic diseases and disorders. The simplicity, utility, and robustness of this technology have revolutionized genomic and epigenome editing for research and translational medicine. Furthermore, initial success has inspired efforts to discover new systems for targeting and manipulating DNA and RNA on the epigenetic level. The rational design and construction of different types of designer molecules paired with viral-mediated gene-to-cell transfers, specifically using lentiviral vectors (LVs) and adeno-associated vectors (AAVs) discussed in detail in this review, as they provide an attractive means for the development of innovative approaches to treat genetic diseases and disorders in need. Notwithstanding the speedy progress of CRISPR/Cas-based gene therapy products, including those based on the epigenome-based editing, multiple challenges outlined by undesirable off-target effects, oncogenicity and other virus-induced toxicities could compromise the successful translation of these new modalities. We believe, that circumventing these challenges will be essential for advancing CRISPR/Cas-based tools towards clinical use in gene and cell therapies. Nevertheless, the speedy evolution and the improvements of CRISPR/Cas9 technologies provide us with a reasonable belief, that in the near future, we will be able to treat and even prevent the most severe and so far, untreatable hereditary diseases and disorders using the technology. In fact, significant advances and positive prospects of the CRISPR/Cas, discussed in this review, may support the optimism and hope that the innovative technology will greatly contribute towards the development of novel treatments for various human hereditary diseases including blood disorders, cystic fibrosis, Alzheimer’s, Huntington’s, Parkinson’s diseases and many others.
